# Water and Collagen Content Are High in Pancreatic Cancer: Implications for Quantitative Metabolic Imaging

**DOI:** 10.3389/fonc.2020.599204

**Published:** 2021-01-27

**Authors:** Marie-France Penet, Samata Kakkad, Flonné Wildes, Zaver M. Bhujwalla

**Affiliations:** ^1^Division of Cancer Imaging Research, The Russell H. Morgan Department of Radiology and Radiological Science, The Johns Hopkins University School of Medicine, Baltimore, MD, United States; ^2^Sidney Kimmel Comprehensive Cancer Center, The Johns Hopkins University School of Medicine, Baltimore, MD, United States; ^3^Department of Radiation Oncology and Molecular Radiation Sciences, The Johns Hopkins University School of Medicine, Baltimore, MD, United States

**Keywords:** pancreatic cancer, total choline, MRS, collagen, water content

## Abstract

In magnetic resonance metabolic imaging, signal from the water content is frequently used for normalization to derive quantitative or semi-quantitative values of metabolites *in vivo* or *ex vivo* tumors and tissues. *Ex vivo* high-resolution metabolic characterization of tumors with magnetic resonance spectroscopy (MRS) provides valuable information that can be used to drive the development of noninvasive MRS biomarkers and to identify metabolic therapeutic targets. Variability in the water content between tumor and normal tissue can result in over or underestimation of metabolite concentrations when assuming a constant water content. Assuming a constant water content can lead to masking of differences between malignant and normal tissues both *in vivo* and *ex vivo*. There is a critical need to develop biomarkers to detect pancreatic cancer and to develop novel treatments. Our purpose here was to determine the differences in water content between pancreatic tumors and normal pancreatic tissue as well as other organs to accurately quantify metabolic differences when using the water signal for normalization. Our data identify the importance of factoring the differences in water content between tumors and organs. High-resolution proton spectra of tumors and pancreatic tissue extracts normalized to the water signal, assuming similar water content, did not reflect the significantly increased total choline observed in tumors *in vivo* without factoring the differences in water content. We identified significant differences in the collagen 1 content between Panc1 and BxPC3 pancreatic tumors and the pancreas that can contribute to the differences in water content that were observed.

## Introduction

Most pancreatic cancers are histologically classified as pancreatic ductal adenocarcinoma (PDAC), and have a 5-year survival rate of less than 5% (1). PDAC is an aggressive and lethal disease that develops relatively symptom-free and is therefore advanced at the time of diagnosis. Its poor prognosis is due to a combination of late-stage diagnosis and limited response to chemotherapy and radiotherapy. The limited response of PDAC to chemotherapy arises, in part, from the strong desmoplastic stroma that limits delivery of therapeutic agents (2). The absence of early symptoms and effective treatments has created a critical need for identifying new noninvasive biomarkers and therapeutic targets. Magnetic resonance spectroscopic imaging (MRSI) and magnetic resonance spectroscopy (MRS) are being evaluated in the diagnosis of several solid malignancies including brain, prostate and breast cancer (3). A hallmark of most solid tumors is the detection of elevated levels of phosphocholine (PC) and total choline (tCho) (4). tCho, which is usually seen as a single peak *in vivo*, consists of three choline-containing metabolites that can be resolved through high-resolution ^1^H MRS into three resonance peaks, namely PC, glycerophosphocholine (GPC) and free choline (Cho). We previously observed elevated levels of tCho in several pancreatic cancer cell lines and tumor xenografts (5). However, initial single voxel studies performed in humans suggest that the tCho signal normalized to water may be relatively high in normal pancreas compared to PDAC (6, 7). Our initial high-resolution *ex vivo* ^1^H MR spectra of tumors and pancreatic tissue normalized to the water signal, assuming similar water content, also did not reflect the significantly increased tCho observed *in vivo*. To assess the importance of tissue heterogeneity, we performed *ex vivo* ^1^H MRSI of tumor tissues, and normal pancreas. We determined if differences in water content caused the discrepancy between our previous *in vivo* observations (5), and the *in vitro* results. Here, we measured the water content in human pancreatic cancer xenografts, in mouse pancreas, and in mouse lungs, liver, heart, kidney and muscle.

Pancreatic tumors are known for their desmoplastic stroma. The extracellular matrix (ECM) plays a critical role in tumor progression and invasion. Stromal collagen 1 (Col1) is a major structural component of the ECM in tumors. PDAC associated desmoplasia can lead to a 3-fold increase in collagen deposition compared to normal tissue (8) that may also alter water content. The collagen triple helix is a unique protein motif that requires water to stabilize its conformation and assembly (9). To assess differences in Col1 fiber in our pancreatic models, we used second harmonic generation (SHG) microscopy (10).

Our data demonstrate the importance of determining differences in water content between tumors and organs. We identified differences in Col1 fibers between the tumors and pancreas that may contribute to the differences in water content between these tissues.

## Material and Methods

### Cell Lines and Tumor Implantation

Human pancreatic cancer cell lines BxPC3 and Panc1 derived from primary adenocarcinoma were obtained from ATCC (American Tissue Culture Collection, Manassas, VA). Cells were cultured in DMEM (Sigma, St. Louis, MO) with 10% fetal bovine serum (FBS), 25 mM glucose and 4 mM glutamine, in standard cell culture incubator conditions at 37°C in a humidified atmosphere containing 5% CO_2_. Subcutaneous and orthotopic tumor implantation was performed as previously described (11). Briefly, viable tumor pieces of ∼1 mm^3^ harvested from subcutaneous tumors were implanted into the pancreas of anesthetized male severe combined immunodeficient (SCID) mice *via* a left subcostal incision of ∼7 mm. Similar sized tumor pieces were subcutaneously implanted in a second set of SCID mice. The tumor pieces used for the implantation were obtained by inoculating 2 x 10^6^ cells suspended in 0.05 ml of Hanks balanced salt solution in the flank of SCID male mice. All surgical procedures and animal handling were performed in accordance with protocols approved by the Johns Hopkins University Institutional Animal Care and Use Committee, and conformed to the Guide for the Care and Use of Laboratory Animals published by the NIH.

### Tissue Water Content

The water content of the tumors, pancreas, lungs, liver, kidney, heart, spleen and muscle was estimated by comparing the wet weight to the dry weight. Tissue wet weights were measured immediately after excision from euthanized mice. The organs were then freeze-clamped and lyophilized for 72h to obtain the corresponding tissue dry weights. The fractional water content was determined as (wet weight-dry weight)/wet weight. We also calculated the ratio of the wet weight to dry weight for all the organs (Healthy organs n=4, Panc1 tumors n=2 (orthotopic n=1, subcutaneous n=1), BxPC3 tumors n=5 (orthotopic n=3, subcutaneous n=2)).

### *Ex Vivo* Magnetic Resonance Spectroscopic Imaging

Mice were sacrificed 4 weeks post-implantation. Orthotopic Panc1 and BxPC3 tumors, subcutaneous Panc1 and BxPC3 tumors, pancreas and thigh muscle were placed in a 50 ml Falcon tube filled with 0.5% agarose. The pancreatic and muscle tissues originated from the subcutaneous tumor-bearing mouse. To be able to visualize all the tissues within one slice during the acquisition, we first poured about 10–15 ml of agarose in the tube that was put on ice for the agarose to solidify. We then carefully placed the organs on top of the first layer of agarose, and slowly added 10 more ml of agarose that was allowed to solidify on ice. To scan the embedded tissues, the tube was placed in a 30 mm diameter volume coil with the tissues located at the center. Data were acquired on a 9.4T horizontal Bruker spectrometer (Bruker Biospin Corp., Billerica, MA). First, we acquired 12 consecutive 1 mm thick T_1_-weighted images to optimize the selection of 4 mm and 1 mm thick slices. Before acquiring the MR spectroscopic images, we acquired T_1_-weighted anatomic images of the selected 4 mm and 1 mm slices. The slice geometry was used for magnetic resonance spectroscopic imaging (MRSI) acquisition and analysis. We acquired water-suppressed MRSI, using VAPOR water suppression and the following parameters: echo time of 135 ms, repetition time of 1,500 ms, field of view of 1.6 cm x 1.6 cm, phase encode steps of 16, number of scans (NS) 4 for the 4 mm thick slice, 12 for the 1 mm thick slice, block size 2048, and sweep width of 10,000 Hz. Unsuppressed water used for normalization was acquired with MRSI from the same slices, with a TE of 20 ms and a NS of 2. All other parameters were kept similar. Spectroscopic images of the tCho signal at 3.2 ppm and the water signal at 4.7 ppm were generated from the MRSI data sets using an in-house IDL program and analyzed using the freeware program ImageJ. The images shown in the center row in **Figure 2**, obtained using the Bruker Paravision display software, are tCho intensity maps overlaid on anatomic T_1_ weighted images. The tCho maps were not quantitative and used heavy thresholding. The images shown on the right in **Figure 2** were obtained using an IDL software developed to quantify tCho concentration in each voxel. To improve sensitivity, anodization windows were implemented in both spectral and spatial dimensions that resulted in smoothing of reconstructed metabolite images.

We corrected the concentration values using a correction factor of 1.18 for Panc1, and 1.19 for BxPC3, obtained from the ratio of the water content of the tumor to the water content of the pancreas. Sample sizes were: Pancreas n=5, Muscle n=4, Panc1 tumors n=4 (orthotopic n=2, subcutaneous n=2), BxPC3 tumors n=5 (orthotopic n=3, subcutaneous n=2).

### Magnetic Resonance Spectroscopy of Dual Phase Extracts

Tumors were excised with half the tumor used for extraction to acquire high-resolution *ex vivo* ^1^H MR spectra and the other half fixed in formalin for histology and to detect Col1 fibers. Tumor and normal pancreas extracts were obtained using a dual-phase extraction method with methanol/chloroform/water (1/1/1) (12). Normal pancreatic tissue and tumor tissue were freeze-clamped and ground to powder. Ice-cold methanol was added, and the tissue extract samples were homogenized, after which chloroform, followed by ice-cold water were added. Tissue extract samples were kept at 4°C overnight for phase separation. Samples were then centrifuged for 30 min at 15,000 g at 4°C to separate the phases. The water/methanol phase containing the water-soluble metabolites was treated with chelex (Sigma, St. Louis, MO) for 10 min on ice to remove divalent cations. The chelex beads were removed through filtration. Methanol was then removed by rotary evaporation, and the remaining water phase was lyophilized and stored at −20°C. Water-soluble samples were dissolved in 0.6 ml of D_2_O (Sigma, St. Louis, MO) containing 3-(trimethylsilyl) propionic-2,2,3,3,-d4 acid (TSP) (Sigma, St. Louis, MO) as an internal concentration standard. Fully relaxed ^1^H MR spectra of the extracts were acquired on a Bruker Avance 500 spectrometer operating at 11.7 T (Bruker BioSpin Corp., Billerica, MA) using a 5-mm HX inverse probe, and the following acquisition parameters: 30° flip angle, 6,000 Hz sweep width, 11 s repetition time, time-domain data points of 32 K, and 128 transients (12). Spectra were analyzed using Bruker XWINNMR 3.5 software (Bruker BioSpin Corp., Billerica, MA). Integrals of the metabolites of interest were determined, and metabolite peak integration values from ^1^H spectra were compared to the internal standard. Values were first normalized to the wet weight of the tissue, then corrected using a factor of 1.82 for Panc1, and 1.85 for BxPC3, obtained from the ratio of the wet to dry weight of the tumor to the wet to dry weight of the pancreas (BxPC3 n = 3; Panc1 n = 3; normal pancreas n = 4).

### Histology and Second Harmonic Generation Microscopy

For histology, the formalin fixed tissues were paraffin embedded, sectioned at a thickness of 5 µm and stained with H&E. Col1 fiber images were acquired from tumor H&E sections using SHG microscopy on a multiphoton Olympus Laser Scanning FV1000 MPE microscope (Olympus Corp., Center Valley, PA, USA) with a 25Xw/1.05XLPLN MP lens. The second harmonic signal was detected at a wavelength of 430 nm following excitation at 860 nm. SHG is an intrinsic signal that arises from non-centrosymmetric molecular structures such as Col1 fibers (10). The SHG analysis was done as previously described (10). Briefly, we used our home-built software in MATLAB R2017b (The MathWorks, Natick, MA, USA) to quantify percent fiber volume and inter-fiber distance. Ten random fields of view (FOVs) were selected and mean and standard error was calculated for each group of pancreatic tumors and pancreatic tissue (BxPC3 n = 4; Panc1 n = 2; normal pancreas n = 2).

### Statistical Analysis

Statistical analyses were performed using GraphPad Prism 8 software (GraphPad Software, Inc., San Diego, CA, USA). To determine the statistical significance of the quantified data between multiple groups, One-way ANOVA was performed, followed by multiple comparisons between each group. *P* values ≤ 0.05 were considered significant unless otherwise stated.

## Results

**Figure 1:**
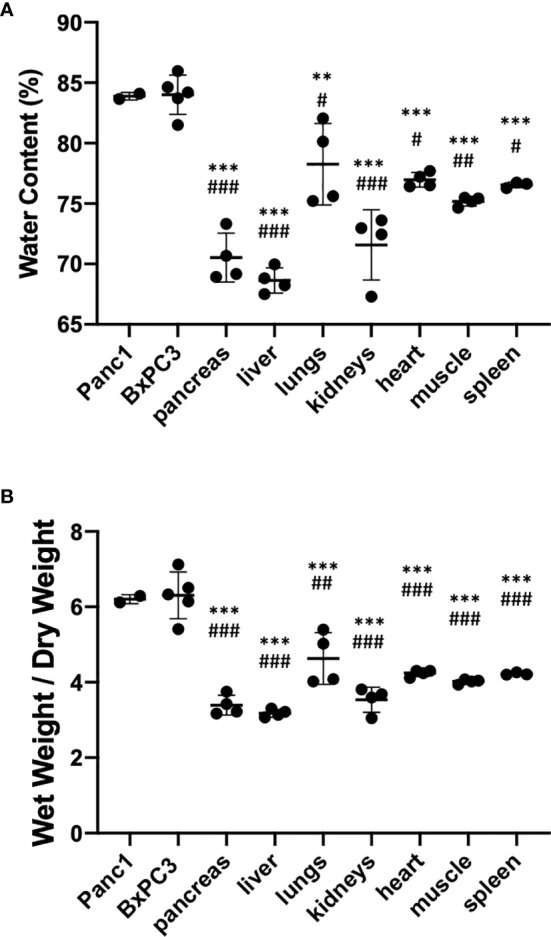
**(A)** Percent water content and **(B)** wet weight over dry weight ratio in different tissues identifying higher water content in tumors compared to the pancreas and other organs. Healthy organs n=4, Panc1 tumors n=2 (orthotopic n=1, subcutaneous n=1), BxPC3 tumors n=5 (orthotopic n=3, subcutaneous n=2). Values represent Mean +/− SD. One-way ANOVA: *P* < 0.0001. Dunnett’s multiple comparison test: ^#^*p* < 0.01, ^##^*p* < 0.001, ^###^*p* < 0.0001 for comparison to Panc1 tumors; *p < 0.01, ^**^p < 0.001, ^***^p < 0.0001 for comparison to BxPC3 tumors.

To assess the water content in tumor, pancreas, liver, lungs, kidney, heart, muscle, and spleen, we weighed the different tissues before and after lyophilization. The percent water content in the different tissues and organs is shown in **Figure 1A**. We also measured the ratio of wet weight to dry weight for each organ (**Figure 1B**). Significantly higher water content and wet weight to dry weight ratios were measured in Panc1 and BxPC3 tumors as compared to other organs, including the pancreas. The ratio of the tumor water content to the pancreas water content was used as a correction factor for the ^1^H MRSI data. In the MRSI data, the tCho concentration is calculated based on the water content, assuming a constant water concentration of water. Therefore, the correction factor used was obtained from the ratio of the water content of the tumor (Panc1: 83.88%, BxPC3: 84.00%) to the water content of the pancreas (70.53%) (*i.e.* 1.18 (Panc1) and 1.19 (BxPC3)).

The tumor to pancreas wet weight to dry weight ratio was used as a correction factor for the high-resolution extracts. Here, values measured with ^1^H MRS were normalized to the wet weight of the tissue. To correct for differences in water content, we used the ratio of the wet to dry weight of the tumor (Panc1: 6.20, BxPC3: 6.30) to the wet to dry weight of the pancreas (3.41) (*i.e.* 1.82 (Panc1) and 1.85 (BxPC3)).

**Figure 2:**
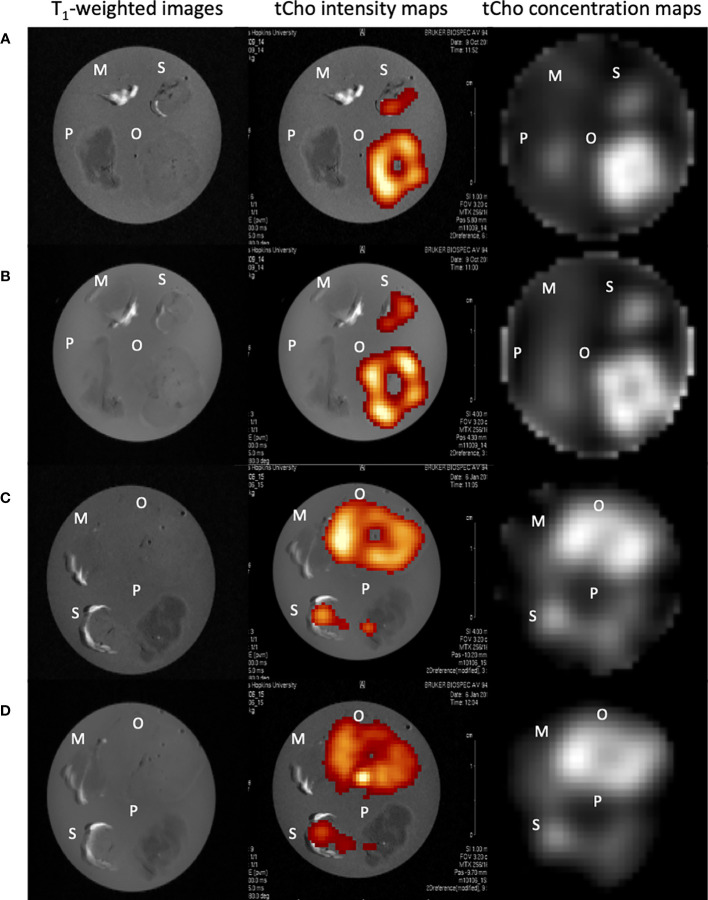
Representative *ex vivo* images of muscle, pancreas, orthotopic and subcutaneous Panc1 tumors **(A, B)**, muscle, pancreas, orthotopic and subcutaneous BxPC3 tumors **(C, D)**. T_1_-weighted images, tCho intensity map, tCho concentration map of 1 mm thick slices **(A, C)** and of 4 mm thick slices **(B, D)** are shown. M-muscle; O-orthotopic tumor; P-pancreas; S-subcutaneous tumor.

*Ex vivo* ^1^H MRSI was performed on orthotopic and subcutaneous Panc1 and BxPC3 tumors, and the pancreas and muscle. Representative images are shown in **Figure 2**. MRSI data were acquired on a 1 mm thick slice (**Figures 2A, C**), and on a 4 mm thick slice (**Figures 2B, D**). Intensity and concentration maps of tCho concentration maps acquired *ex vivo* confirmed high levels of tCho in Panc1 and BxPC3 orthotopic tumors, followed by subcutaneous tumors. A much lower signal was detected in the pancreas, with none in the muscle (**Figure 2**). The maps also confirmed the heterogeneous tCho distribution within the tumors.

**Figure 3:**
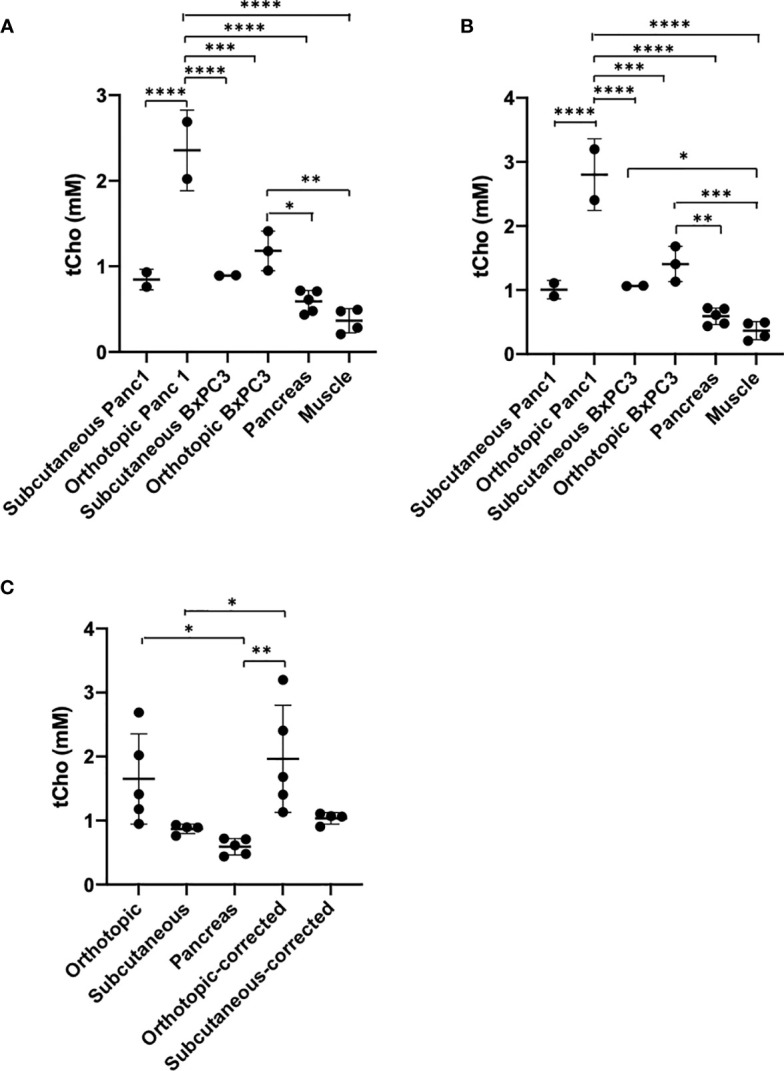
**(A)** tCho concentration quantification from the *ex vivo* 4 mm thick ^1^H MRSI maps without correction. Pancreas n=5, Muscle n=4, Panc1 tumors n=4 (orthotopic n=2, subcutaneous n=2), BxPC3 tumors n=5 (orthotopic n=3, subcutaneous n=2). **(B)** tCho concentration quantification from the *ex vivo* 4 mm thick ^1^H MRSI maps after correction. **(C)** tCho concentration in both subcutaneous and orthotopic tumor types averaged before and after correction (subcutaneous tumors: n=4; orthotopic tumors and pancreas: n=5). Values represent Mean +/− SD. One-way ANOVA *P*<0.0001 **(A, B)** *P* < 0.004 **(C)**. Dunnett’s multiple comparison test: *p < 0.05, **p < 0.01, ***p < 0.001, ****p < 0.0001.

Quantifications of tCho in the different tissues calculated from the *ex vivo* ^1^H MRSI data are summarized in **Figure 3**. Orthotopic tumors showed significantly higher levels of tCho compared to subcutaneous tumors. tCho concentrations were higher in the tumors as compared to pancreas and muscle. Panc1 orthotopic tumors presented significantly higher levels of tCho as compared to orthotopic BxPC3 tumors. Taking into account the differences in water content between the pancreas and tumor, we corrected the concentration values using a correction factor of 1.18 and 1.19 respectively for Panc1 and BxPC3, obtained from the ratio of the water content of the tumor to the water content of the pancreas (**Figures 3B, C**). Correcting the values strengthened the significant difference between the tumors and the pancreas.

**Figure 4:**
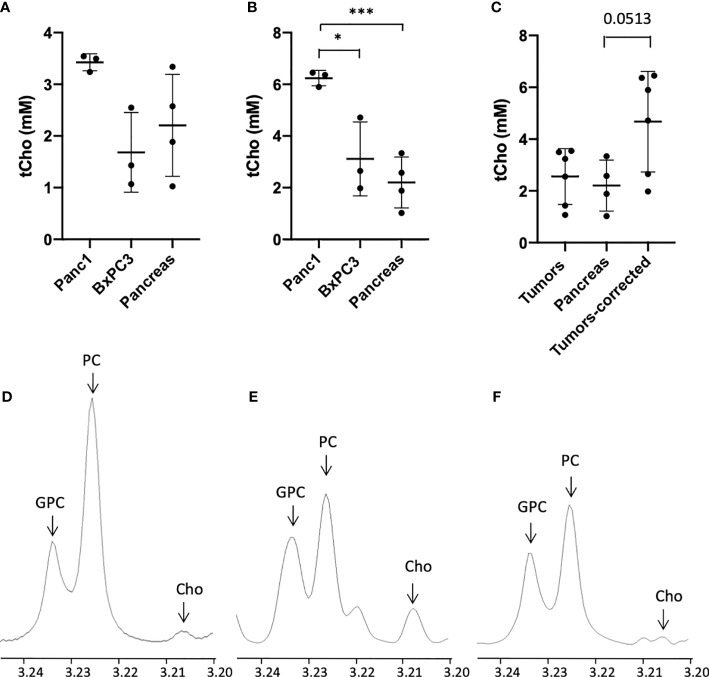
tCho concentrations in mM derived from the water phase of Panc1, and BxPC3 orthotopic tumor extracts with high-resolution ^1^H MRS (tCho=Cho+PC+GPC) compared to normal adjacent pancreas before **(A)** and after **(B)** water content correction, (n=3). **(C)** tCho concentrations in mM averaged for the orthotopic tumors before and after correction as compared to normal pancreas (n=12). Representative ^1^H MR spectra of orthotopic Panc1 tumor **(D)**, orthotopic BxPC3 tumor **(E)** and normal pancreas **(F)** (x-axis, chemical shift in ppm). Values represent Mean +/- SD. One-way ANOVA *P* = 0.06 **(A)** *P* < 0.004 **(B)** *P* < 0.04 **(C)** Dunnett’s multiple comparison test: *p < 0.05, ***p < 0.005.

We then performed dual phase extraction of orthotopic Panc1, and BxPC3 tumors, as well as adjacent normal pancreas, and acquired high-resolution ^1^H MRS on the extracts to measure tCho (Cho + PC + GPC) concentrations. The values were initially normalized to the wet tissue weight, (**Figure 4A**). We observed a trend to higher tCho in tumor as compared to the adjacent normal pancreas. After factoring in differences in water content, the difference between tumors and pancreas was significant for Panc1 tumors (**Figure 4B**). We used a correction factor of 1.82 and 1.85 respectively for Panc1 and BxPC3, obtained from the ratio of the wet to dry weight of the tumor to the wet to dry weight of the pancreas. When combining the 2 tumor types, the difference between tumors and pancreas became significant after correction (**Figure 4C**).

**Figure 5:**
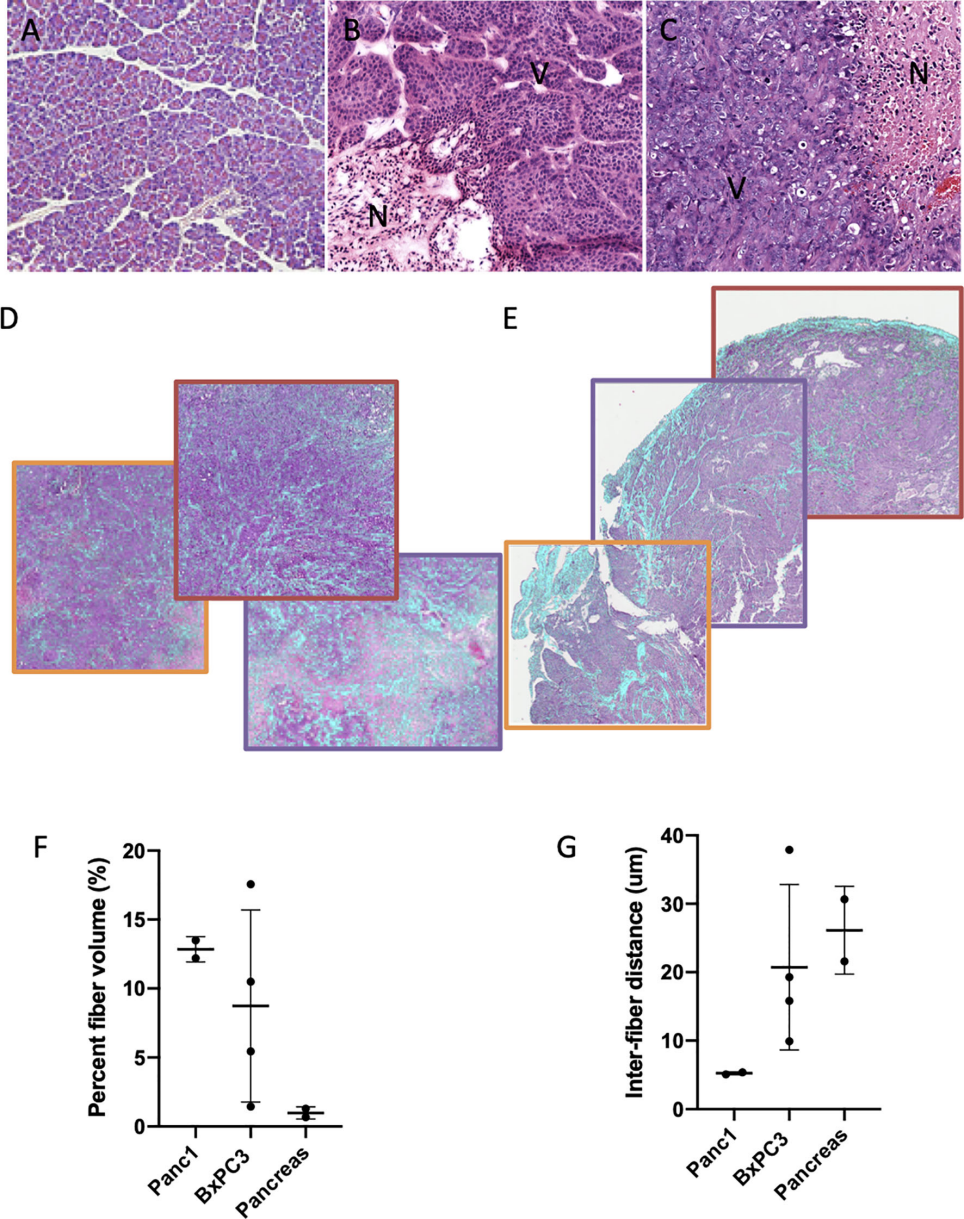
H&E-stained sections of normal pancreas **(A)**, orthotopic BxPC3 tumor **(B)**, orthotopic Panc1 **(C)**. (N-necrotic area, V-viable tissue). Second harmonic generation (SHG) images overlaid with H&E-stained sections of orthotopic BxPC3 tumor **(D)**, orthotopic Panc1 tumor **(E)** in three different fields of view. Quantification of fiber volume **(F)** and inter fiber distance **(G)** in normal pancreas, orthotopic BxPC3 tumor, and orthotopic Panc1 tumor. Values represent Mean +/- SD.

Differences in tCho concentration were observed between each tumor type, with Panc1 showing the highest levels. The analysis of H&E stained tumor sections revealed differences, as shown in **Figure 5**, between Panc1 and BxPC3. Necrotic areas were observed in both tumor types, which could partly explain the heterogeneity of tCho signal observed (**Figure 2**), highlighting the importance of acquiring ^1^H MRSI instead of single voxel MRS. Tumor heterogeneity can also complicate biopsy sample analysis and extract analysis, if only part of the tumor is used for analysis. Col1 fiber content was analyzed in the histologic sections using SHG microscopy. Normal pancreas had about 1% of Col1 fiber volume. Fibrotic stroma with Col1 fibers surrounding the cancer cells were observed in both Panc1 and BxPC3 tumors, with a different organization profile (**Figures 5D, E**) between the two tumor types. BxPC3 tumors tended to have less fibers than Panc1 tumors, with higher distance between the fibers (**Figure 5F**). Tile scan analysis was also performed on the tumor sections, giving identical results as the random 10 FOVs (data not shown).

## Discussion

We have previously shown heterogeneous tCho distribution *in vivo* using ^1^H MRSI in subcutaneously and orthotopically implanted human pancreatic tumors (5). In the present study, the heterogeneous distribution was confirmed *ex vivo*. We also confirmed the higher tCho content in Panc1 tumors as compared to BxPC3, as previously described (5). To quantify ^1^H MRSI, the water signal is typically used as a reference. Our data identified a significantly lower water content in normal tissues including the pancreas compared to subcutaneous and orthotopic pancreatic tumors, highlighting the importance of factoring in differences in water content when quantifying metabolites for comparison across different tissue types. Similarly, quantification of high resolution ^1^H MRS is usually normalized to the tissue wet weight, and the water content should be taken into account when comparing different tissues characterized by different water content. Our results also support the use of ^1^H MRSI that provides a tCho map rather than single voxels ^1^H MRS to address heterogeneities in the pancreas and in pancreatic tumors.

The differences in water content that we observed between the tumors and the normal organs are consistent with previous studies. Water content in pancreatic tumors was reported to be higher than 80%, while the water content of normal pancreas was found to be 70% in tumor models, that included the KPC model, as well as allografts and xenografts (13). Tumor models of other cancers have similar results with the water content higher than 80%, while normal tissues, including brain, heart, kidney and liver were under 70% (14). In studies with murine tumors, the water content was 84% in Panc02 pancreatic tumors, 83.5% in recticular cell sarcoma M5076 tumors, and 79% in RIF-1 tumors (15). Interestingly, we did not observe any differences between the water content of subcutaneous and orthotopic tumors. Also, both tumor types investigated showed similar water content.

A higher level of tCho was previously reported in pancreatic tumors compared to normal pancreas in a transgenic pancreatic neuroendocrine tumor mouse model (16). These preclinical results were confirmed by *in vitro* ^1^H MRS of human biopsies. The studies demonstrated the potential of using tCho detection by ^1^H MRS to identify pancreatic tumors *in vivo*. Similar to our study, while GPC, PC and Cho were detected in the normal pancreas, significantly higher levels were measured in the tumor compared to normal tissue. In this neuroendocrine tumor model, a 3-fold increase in GPC, PC and Cho was observed in the tumor compared to the normal tissue. In the same study, analysis of human biopsies showed higher PC levels compared to GPC levels, and a higher concentration of tCho in tumor as compared to normal pancreas (16).

In another study, early pancreatic lesion metabolite profiles were investigated in a Kras (p48-Cre;LSL-Kras^G12D^) mouse model and in human biopsies using ^1^H high resolution magic angle spinning (HR-MAS) MRS (17). Analysis of human PDAC samples revealed a reduction of lipids, and an increase of lactate and taurine in the tumors, but unchanged tCho levels. A limited number of cases and tumor heterogeneity may have influenced these biopsy results. Additionally, concentrations were calculated after normalization to an electronic reference, and to the sample mass. In the Kras (p48-Cre;LSL-Kras^G12D^) mouse model, a decrease of PC was observed (17). The Kras (p48-Cre;LSL-Kras^G12D^) model is characterized by the development of early pre-neoplastic pancreatic intraepithelial neoplasia (PanIN) lesions, but rarely develops into late PDAC tumors. This may partly explain the differences between these results and our data.

While we observed the presence of Cho, PC and GPC in normal pancreas, we also observed differences in water content between normal pancreas and tumor tissue. Assuming a similar water content between normal pancreas and tumor tissue can significantly impact the accuracy of the tCho concentration, since the water content is significantly different between the two tissues. *Ex vivo* ^1^H MRSI data revealed higher concentration of tCho in tumor tissues compared to pancreas, especially in the orthotopically implanted tumors. The tCho maps also revealed the importance of acquiring ^1^H MRSI instead of single voxel ^1^H MRS because of the heterogeneity of the tCho distribution within the tumor tissue. The tCho heterogeneity, observed in both Panc1 and BxPC3 tumors, is due partly to the presence of necrotic area, but also to the variable density of cancer cells within the ECM, and in fibrotic area, as seen on the histologic sections. The use of ^1^H MRSI that provides a tCho map rather than signal from a single voxel is important to address metabolic heterogeneities in pancreatic cancers, and in the pancreas. Investigations comparing spectra obtained from normal and malignant pancreatic regions will require precise placement of voxels in viable non-necrotic tumor regions, elimination of motion-related effects, and accurate quantitation of metabolites.

Due to the heterogeneous nature of the desmoplastic reaction in PDAC, localized biopsies can lead to a misrepresentation of the extent of fibrosis in the tumor. The potential of using either water apparent diffusion coefficient (ADC) measurements or magnetization transfer (MT) MRI to noninvasively assess tumor fibrosis has been recently explored (8). While ADC is based on water molecule diffusion, MT MRI is based on the exchange of magnetization between subpopulations of free water protons and water protons bound to tissue macromolecules. MT contrast can be sensitive to tissue collagen concentration and MTR values were shown to be higher in BxPC3 tumors, consistent with their higher fibrosis levels (8). ADC values were identified as significant prognostic factors in pancreatic cancer patients (18).

SHG microscopy analysis of Panc1 and BxPC3 orthotopic tumors revealed differences in Col1 fiber patterns, and increased fiber content as compared to normal pancreas. Differences in Col1 fiber pattern have been previously described in subcutaneous Panc1, BxPC3 and Capan-1 tumors (8), with a higher level of fibrosis and associated collagen deposition, as measured with Trichrome Masson, in BxPC3 tumors compared to Panc1 and Capan-1 tumors. This study performed in subcutaneous models did not include normal pancreas. In our studies the most significant difference was between collagen content in tumors compared to the normal pancreas. Differences in ECM and collagen content and organization can contribute to differences in the water content of tissues. Water binds to matrix proteins, including collagen (19) and plays a critical role in stabilizing collagen structure. The mechanical properties of collagen change with hydration. The concentration of collagen fibers in pancreatic tumors correlates with the total tissue pressure (20). Tumors are characterized by biomechanical alterations that include accumulation of solid stress, ECM stiffening and increased interstitial fluid pressure due to leaky blood vessels, dysfunctional lymphatics and a dense interstitial ECM (21). The dense ECM characterizing PDAC includes high concentration of interstitial hyaluronan (HA) (13). HA is a high-molecular weight polysaccharide that forms networks with proteoglycans in the ECM. It attracts water causing tissue swelling (22). Collagen encapsulates HA allowing the collagen microfibrillar network to restrain the intrinsic swelling pressure of HA (22). HA can bind up to 15 molecules of water per disaccharide unit, due to its high negative charge that contributes to its ability to complex large amount of water (13). Our results support further investigation of the role of the ECM and Col1 fibers in influencing the water content of pancreatic cancers.

Taken together our data identified significant differences in water content between pancreatic cancer tissue and normal tissue including the pancreas, as well as the differences in Col1 fiber content between these tissues. Our data highlight the importance of including the difference in water content when quantifying metabolic differences between normal and malignant tissue. The significant increase of tCho in pancreatic cancer compared to normal pancreatic tissue is consistent with previous studies that have identified an increase of tCho in pancreatic cancer supporting its investigation in the detection of pancreatic cancer.

## Data Availability Statement

The raw data supporting the conclusions of this article will be made available by the authors, without undue reservation.

## Ethics Statement

The animal study was reviewed and approved by the Johns Hopkins University Institutional Animal Care and Use Committee.

## Author Contributions

Conception and design: M-FP and ZB conceptualized and designed the study. M-FP, FW, and SK collected and assembled the data. M-FP, SK, and ZB analyzed and interpreted the data. All authors contributed to the article and approved the submitted version.

## Funding

This work was supported by NIH R01CA82337 and R01CA193365.

## Conflict of Interest

The authors declare that the research was conducted in the absence of any commercial or financial relationships that could be construed as a potential conflict of interest.
